# Impact of Coffee Consumption on Cardiovascular Health

**DOI:** 10.31486/toj.22.0073

**Published:** 2023

**Authors:** Michael F. Mendoza, Ralf Martz Sulague, Therese Posas-Mendoza, Carl J. Lavie

**Affiliations:** ^1^ The Gayle and Tom Benson Cancer Center, Ochsner Clinic Foundation, New Orleans, LA; ^2^ School of Health, Georgetown University, Washington, DC; ^3^ Department of Rheumatology, Ochsner Clinic Foundation, Covington, LA; ^4^ Department of Cardiology, Ochsner Clinic Foundation, New Orleans, LA; ^5^ The University of Queensland Medical School, Ochsner Clinical School, New Orleans, LA

**Keywords:** *Atrial fibrillation*, *cholesterol*, *coffee*, *coronary disease*, *diterpenes*, *heart failure*, *hypertension*, *lipids*, *phenolic acid*

## Abstract

**Background:** Coffee is a widely available beverage that is enjoyed by individuals of many cultures. The publication of new studies prompts a review of the clinical updates regarding the association between coffee consumption and cardiovascular disease.

**Methods:** We present a narrative review of the literature related to coffee consumption and cardiovascular disease.

**Results:** Recent (2000-2021) studies have shown that regular coffee consumption is associated with a decreased risk of developing hypertension, heart failure, and atrial fibrillation. However, results are inconsistent with regard to coffee consumption and risk of developing coronary heart disease. Most studies show a J-shaped association, wherein moderate coffee consumption resulted in decreased risk of coronary heart disease and heavy coffee consumption resulted in increased risk. In addition, boiled or unfiltered coffee is more atherogenic than filtered coffee because of its rich diterpene content that inhibits bile acid synthesis and ultimately affects lipid metabolism. On the other hand, filtered coffee, which is essentially devoid of the aforementioned compounds, exerts antiatherogenic properties by increasing high-density lipoprotein–mediated cholesterol efflux from macrophages through the influence of plasma phenolic acid. As such, cholesterol levels are principally influenced by the manner of coffee preparation (boiled vs filtered).

**Conclusion:** Our findings suggest that moderate coffee consumption leads to a decrease in all-cause and cardiovascular-related mortality, hypertension, cholesterol, heart failure, and atrial fibrillation. However, no conclusive relationship between coffee and coronary heart disease risk has been consistently identified.

## INTRODUCTION

Coffee, one of the most popular beverages in the world, is widely enjoyed regardless of individuals’ ethnicity, sex, or cultural background because of its psychoactive effects, including mental alertness, vigilance, reaction time, and productivity. Its accessibility and extensive consumption have generated great interest regarding its overall impact on health. Concerns have been raised about the ability of coffee to induce cardiac arrhythmias and increase blood pressure.^1,2^ In the 1960s, coffee consumption was labeled as a cardiovascular risk factor associated with coronary heart disease.^3^

Americans, similar to Europeans, are estimated to consume approximately 5.1 kg of coffee per person per year.^4^ The amount of caffeine, a major component of coffee, varies between 30 mg and 175 mg in a single home-prepared cup of coffee.^4^ The stimulant effect of caffeine is caused by the antagonism of adenosine receptors, effectively inhibiting the effects of adenosine, a well-known inhibitory neuromodulator. However, caffeine is not a completely benign compound, as it can cross the placenta during pregnancy and may potentially cause conditions such as spontaneous abortion and impaired fetal growth. Therefore, coffee consumption for females who are pregnant or planning to become pregnant should be limited to a moderate level that does not exceed 300 mg of caffeine per day.^5^

Given the publication of recent (2000-2021) studies regarding coffee consumption, we review clinical updates regarding the impact of coffee consumption on cardiovascular health. This review focuses on the association between coffee consumption and cardiovascular disease–related mortality and morbidity.

## METHODS

We conducted a literature search of the PubMed/MEDLINE database for relevant articles using a combination of key terms such as “coffee,” “coffee consumption,” “cardiovascular diseases,” “hypertension,” “cholesterol,” “myocardial infarct,” and “atrial fibrillation.” We included studies that investigated the association of coffee consumption and various cardiac conditions. This narrative review synthesizes findings from previous research and recent studies on the topic.

## COFFEE CONSUMPTION AND HYPERTENSION

A 1999 meta-analysis of clinical trials by Jee et al that included 11 eligible studies (n=522) revealed a direct increased relationship between coffee consumption and blood pressure readings after a 56-day median follow-up.^6^ An older cohort study by Jenner et al (1988) that included 340 working males aged 20 to 45 years old who were not on any antihypertensive medication for a 6-year period revealed that systolic and diastolic changes in blood pressure were both positively related to baseline age and weight.^7^ In the Jenner et al study, a reduction in coffee consumption was negatively associated with systolic changes in blood pressure.^7^

However, more recent studies have reported contrasting findings. A large cohort study (n=8,780) of middle-aged adults from Brazil by Miranda et al (2021) showed that those who consumed moderate (1 to 3 cups per day) amounts of coffee had less risk of developing hypertension than those who never or almost never drank coffee (relative risk [RR]=0.82, 95% CI 0.68 to 0.97, *P*=0.018).^8^ Notably, these benefits were only observed in those who had never smoked (RR=0.79, 95% CI 0.64 to 0.98) as opposed to current and former smokers who did not show a statistically significant reduction in relative risk.^8^ In a pilot crossover randomized study by Revuelta-Iniesta and Al-Dujaili (2014), green coffee was found to significantly reduce systolic blood pressure (*P*=0.02) by a mean of 2.65 ± 1.37 mm Hg compared to baseline.^9^

A 2018 review by Fan et al proposed several possible mechanisms underlying these results: (1) the augmentation of cytochrome P450 1A2 (CYP1A2) activity (the main enzyme for metabolizing caffeine), which is inversely correlated with blood pressure levels in coffee drinkers who are nonsmokers; (2) the inhibition of sodium and water reabsorption; and (3) the inhibition of inflammation, oxidative stress, and the renin-angiotensin-aldosterone system (RAAS) via chlorogenic acid, which is found in high concentrations in coffee.^10^ In 2009, the Hypertension and Ambulatory Recording VEnetia STudy (HARVEST) found that the risk of developing hypertension was directly associated with coffee consumption but only in people who possessed the allele CYP1A2 variant for slow metabolizers (59% of the population); whereas in those who were rapid CYP1A2 metabolizers, coffee consumption was inversely related to the risk of developing hypertension.^11^

## COFFEE CONSUMPTION AND CHOLESTEROL

As stated previously, boiled or unfiltered coffee is more atherogenic than filtered coffee because of its diterpene content. Cafestol and kahweol are 2 diterpenes in coffee beverages, with approximately 7.2 mg of each substance per cup of boiled coffee.^12^ However, paper-filtering coffee removes most of these oils, with only about 0.02 mg of each substance retained per cup.^12-14^ Post et al (1997) found that diterpenes inhibit bile acid synthesis which leads to attenuated catabolism of lipids.^15^ The researchers observed that cafestol suppressed cholesterol 7α-hydroxylase and sterol 27-hydroxylase in rat hepatocytes, while kahweol had less suppressive effects.^15^

Because boiled coffee is known to have atherogenic capabilities through cafestol and kahweol, filtered coffee that is virtually devoid of these compounds can exert antiatherogenic properties by increasing high-density lipoprotein–mediated cholesterol efflux from macrophages through the influence of plasma phenolic acid, another major component in coffee beverages. Uto-Kondo et al (2010) concluded that plasma phenolic acid is capable of increasing ATP-binding cassette transporter ABCG1 and scavenger receptor class B type I (SR-BI) expression, which are responsible for high-density lipoprotein–mediated cellular cholesterol efflux.^16^

Furthermore, coffee consumption was observed to have a direct linear relationship with total cholesterol levels.^17^ Researchers later (in 1987 and 1989) found that between unfiltered (boiled) and filtered coffee, only boiled coffee was directly associated with an increase in total cholesterol levels.^18,19^ A crossover, randomized, controlled study by Sarriá et al (2018) of 25 normocholesterolemic and 27 hypercholesterolemic males and females aged 18 to 45 years with body mass index 18 to 25 kg/m^2^ found significant decreases in systolic and diastolic blood pressure (*P*=0.001 and *P*<0.001, respectively) in both groups, as well as in percentage of body fat (*P*=0.001) after 3 servings per day of a green/roasted coffee blend, providing 510.6 mg hydroxycinnamic acids and 121.2 mg caffeine per day, vs a control drink (8 weeks each).^20^ Concurrent significant decreases were seen in leptin (*P*=0.001), plasminogen activator inhibitor 1 (*P*<0.001), and resistin (*P*=0.034) levels, as well as glucose concentration (*P*=0.030), insulin resistance (*P*=0.011), and triglyceride levels (*P*= 0.017) in the 2 groups after coffee consumption, with a notably greater reduction in the hypercholesterolemic cohort (group effect, *P*=0.027).^20^ Currently, no specific recommendations on the type and ideal amount of coffee consumption take advantage of these findings.

## COFFEE CONSUMPTION, CORONARY HEART DISEASE, AND CARDIOVASCULAR DISEASE

Despite evidence that cholesterol levels seem to be increased by boiled coffee consumption,^18,19^ the association with coronary heart disease seems to be different. In a prospective cohort study by Lopez-Garcia et al (2006), groups with varying coffee consumption had insignificant RRs for coronary heart disease.^21^ However, results across studies are inconsistent. In an Italian study by Grioni et al (2015)*,* the consumption of more than 2 cups per day of Italian-style coffee was associated with an increased risk of coronary heart disease.^12^ Additionally, a meta-analysis of 13 case-control and 10 cohort studies by Sofi et al (2007) showed a significant association between high coffee consumption and risk of coronary heart disease in the case-control studies: >4 cups per day (odds ratio [OR] 1.83, 95% CI 1.49 to 2.24, *P*<0.0001) and 3 to 4 cups per day (OR 1.33, 95% CI 1.04 to 1.71, *P*<0.0001).^22^ In the long-term follow-up cohort studies, however, the risk of developing coronary heart disease was insignificant for those drinking >4 cups per day (RR 1.16, 95% CI 0.95 to 1.41, *P*=0.14), 3 to 4 cups per day (RR 1.05, 95% CI 0.90 to 1.22, *P*=0.57), and ≤2 cups per day (RR 1.04, 95% CI 0.90 to 1.19, *P*=0.60).^22^

In a study by Rodriguez-Artalejo and López-Garcia (2018), moderate levels of coffee consumption (3 to 5 cups per day) were associated with a 15% reduction in the risk of cardiovascular disease, and higher levels of intake were not shown to increase risk.^23^ In a study by Zhou and Hyppönen (2019), those who did not drink coffee, those who drank decaffeinated coffee, and heavy coffee drinkers (>6 cups per day) had higher odds of developing cardiovascular disease by 11%, 7%, and 22%, respectively (*P*=0.013) compared to those who consumed 1 to 2 cups of coffee per day.^24^

In a meta-analysis of 17 studies involving 233,617 participants, Mo et al (2018) noted an increase in myocardial infarction among males who consumed >3 cups of coffee per day; this effect was not observed in females.^25^ Inconsistencies have also been seen among elderly males and females. A study by van Woudenbergh et al (2008) revealed a significant reduction in coronary calcification in elderly (mean age of 64 years) females with moderate (3 to 4 cups per day) and high (>4 cups per day) coffee intake compared to those with a daily intake of ≤3 cups.^26^ The investigators speculated that the phytoestrogens in coffee could partly replace estrogen stores in postmenopausal females, leading to a decreased incidence of atherosclerosis. This function of phytoestrogen may explain the lack of protective effect of coffee for atherosclerotic calcification in males.^26^

Cornelis et al (2006) shed some light on why these studies commonly reported a dose-dependent J- or U-shaped curve.^27^ According to their study, the increased risk of coronary heart disease among boiled (unfiltered) coffee consumers is associated with diterpenes that increase cholesterol levels and ultimately the risk of coronary heart disease. However, Cornelis et al noted that the risk of developing myocardial infarction was not obviated by filtering diterpenoids.^27^ Variations in CYP1A2 activity among coffee consumers determined the risk of cardiovascular disease, and because CYP1A2 does not metabolize anything else in filtered coffee other than caffeine, the investigators were led to believe that caffeine is the major component of filtered coffee that increases the risk of myocardial infarction. They proposed that the inhibition of the vasodilatory effects of adenosine may play a role in the development of cardiovascular disease. This hypothesis is noteworthy, as coffee does not increase the risk of hypertension, possibly owing to its other antihypertensive effects (diuresis, RAAS inhibition, and antioxidant properties). Cornelis et al reported that the risk of nonfatal myocardial infarction depended on the ability of an individual to metabolize caffeine.^27^ CYP1A2 accounts for approximately 95% of caffeine metabolism, and great variability in enzyme activity is observed in individuals. The carriers of the variant allele CYPA12*1F are slow caffeine metabolizers, while those who are homozygous with the CYP1A2*1A allele are rapid metabolizers.^27^ This genetic difference could potentially explain the variable results noted across studies. The Cornelis et al study indicated a positive correlation between increased coffee consumption and the risk of nonfatal myocardial infarction but only in those who were slow metabolizers of caffeine.^27^ However, when smoking was involved, the results were once again conflicting because smoking induces CYP1A2 activity, and the magnitude of CYP1A2 induction was less pronounced in those who had the variant allele CYPA12*IF. The risk of myocardial infarction was higher in those who were slow caffeine metabolizers regardless of smoking status. While smoking may appear to decrease the risk of myocardial infarction by induction of the homozygous allele, smokers with the CYP1A2*1A allele do not necessarily bode better than slow metabolizers because smoking is an established independent cardiovascular disease risk factor with other pathologic mechanisms beyond the protective effects of CYP1A2 on caffeine regulation.^27^

The genetic associations identified by Cornelis et al^27^ were not observed in the large prospective analysis by Zhou and Hyppönen (2019).^24^

Results from these studies conflict, and the study designs include many possible confounders. The standardization of coffee preparation and intake is an important issue because the definition of light, moderate, and heavy consumption is not consistent across studies. As such, more studies are needed to reach a solid consensus about the impact of coffee consumption on cardiovascular disease, specifically at higher intake levels.

## COFFEE CONSUMPTION AND CARDIOVASCULAR DISEASE POST MYOCARDIAL INFARCTION

In a study by Silletta et al (2007), no association was found between moderate coffee consumption and cardiovascular disease events in patients who had had a myocardial infarction.^28^ After a mean follow-up of 3.5 years, coffee consumption did not change the risk of developing coronary heart disease, stroke, or sudden cardiac death in those who had previous myocardial infarction. These results are not substantially reliable because the trend finding across categories of coffee consumption was not statistically significant (*P*=0.18).^28^ A meta-analysis by Brown et al (2016) found a statistically significant inverse correlation between coffee consumption and mortality (n=3,271).^29^ Light coffee drinkers (1 to 2 cups per day) vs those who did not drink coffee were associated with a risk ratio of 0.79 (95% CI 0.66 to 0.94, *P*=0.008), while heavy coffee drinkers (>2 cups per day) vs those who did not drink coffee were associated with a risk ratio of 0.54 (95% CI 0.45 to 0.65, *P*<0.00001). Heavy coffee drinkers vs light coffee drinkers had a risk ratio of 0.69 (95% CI 0.58 to 0.83, *P*<0.0001).^29^ These results were supported by a 2020 meta-analysis of 6 prospective studies showing that coffee consumption was associated with lower risk of cardiovascular mortality (hazard ratio [HR] 0.70, 95% CI 0.54 to 0.91, I^2^=0%; 2 studies) and not associated with an increased risk of all-cause mortality (HR 0.85, 95% CI 0.63 to 1.13, I^2^=50%; 3 studies), recurrent myocardial infarction (HR 0.99, 95% CI 0.80 to 1.22, I^2^=0%; 3 studies), or stroke (HR=0.97, 95% CI 0.63 to 1.49, I^2^=39%; 2 studies).^30^

## COFFEE CONSUMPTION AND HEART FAILURE

Data on diet and food intake from the Framingham Heart Study (FHS), the Cardiovascular Health Study (CHS), and the Atherosclerosis Risk in Communities (ARIC) study were studied to identify potential lifestyle and behavioral factors associated with heart failure. Stevens et al (2021) showed an inverse association between coffee consumption and the risk of heart failure in all 3 studies.^31^ Data from the FHS, CHS, and ARIC showed that higher coffee consumption was associated with a lower long-term risk of heart failure.^31^ Mostofsky et al (2012) also observed a statistically significant J-shaped relationship between coffee consumption and heart failure; those who consumed up to 4 servings of coffee per day had a strong inverse relationship with the development of heart failure.^32^ Those who tended to consume coffee at much higher levels (starting at 9 to 10 servings per day) had a potentially higher risk of heart failure.^32^ Mostofsky et al found no evidence that sex, baseline history of myocardial infarction, or diabetes mellitus affected outcomes.^32^ From the Coronary Artery Risk Development in Young Adults study (n=2,735) published by the European Society of Cardiology (2020), low to moderate coffee consumption from young adulthood to middle age was associated with better left ventricular systolic and diastolic function.^33^

Although results seem to be consistent across studies, the potential mechanisms causing reduction in heart failure risk are not well understood. A close look into the biomolecular processes is warranted to strengthen causality.

## COFFEE CONSUMPTION AND ATRIAL FIBRILLATION

Bodar et al (2019) found that consumption of 1 to 3 cups of coffee per day was associated with lower rates of atrial fibrillation.^34^ After adjusting for age, smoking status, alcohol intake, and exercise, those who consumed ≤1 cup per week, 2 to 4 cups per week, and 5 to 6 cups per week were analyzed. In these subgroups, HRs were 0.85 (95% CI 0.71 to 1.02), 1.07 (95% CI 0.88 to 1.30), and 0.93 (95% CI 0.74 to 1.17), respectively. Among those who consumed 1 cup per day, 2 to 3 cups per day, and >4 cups per day, corresponding HRs were 0.85 (95% CI 0.74 to 0.98), 0.86 (95% CI 0.76 to 0.97), and 0.96 (95% CI 0.80 to 1.14), respectively, after adjusting for age, smoking, alcohol intake, and exercise (*P* for nonlinear trend=0.01). These results indicate that those who drank 1 to 3 cups of coffee per day had less risk of atrial fibrillation compared to other intake frequencies.^34^ A meta-analysis of prospective cohorts by Cheng et al (2014) showed an 11% reduction in atrial fibrillation risk for low coffee consumption equivalent to <500 mg of caffeine per day (RR 0.89, 95% CI 0.80 to 0.99, *P*=0.032, I^2^=30.9%, *P*=0.227) and 16% for high coffee consumption equivalent to ≥500 mg of caffeine per day (RR 0.84, 95% CI 0.75 to 0.94, *P*=0.002, I^2^=24.1%, *P*=0.267) after pooling of results from studies with adjustment of potential confounders.^35^ A theory proposed to explain these results is that coffee promotes anti-inflammatory processes through its high levels of antioxidants such as cafestol, polyphenol, trigonelline, chlorogenic acid, and quinine.^34,36^ A meta-analysis of 23 randomized controlled trials by Ali-Hassan-Sayegh et al (2014) reported that antioxidants such as N-acetylcysteine and polyunsaturated fatty acids have a protective role in the prevention of atrial fibrillation after cardiac surgery.^37^ Patients who were infused with these antioxidant compounds experienced less incidence of atrial fibrillation after cardiac surgery.^37^ These results may suggest that coffee consumption, especially in moderation, may induce anti-inflammatory effects that may attenuate the risk of developing atrial fibrillation.

## COFFEE CONSUMPTION AND MORTALITY

A large pool of data indicates that coffee consumption is not associated with increased cardiovascular disease mortality. Results presented by Ding et al (2015) from 3 well-known large prospective cohort studies, the Nurses’ Health Study, the Health Professionals Follow-up Study, and the Nurses’ Health Study-II, showed a nonlinear association between cardiovascular disease mortality and coffee consumption.^38^ In the analysis of the aforementioned cohort studies, moderate coffee consumption was associated with less mortality, and higher levels of consumption did not result in increased deaths after removing the confounding effects of smoking history. As such, the nonlinear relationship was strengthened by removing the residual confounding effect of smoking status. The authors concluded from their analysis that the results extracted from these 3 large cohort studies indicate that coffee consumption can be incorporated into a healthy lifestyle.^38^

The cohort study of Lopez-Garcia et al (2008) revealed an inverse relationship between coffee consumption and all-cause mortality, principally because of a moderate reduction in risk for cardiovascular disease–related mortality in both males (*P* for trend=0.008) and females (*P* for trend <0.001).^39^ The authors noted that a possible mechanism for such an outcome was the strong antioxidant capacity of coffee compounds other than caffeine such as chlorogenic acid, ferulic acid, and p-Coumaric acid. They also noted that other substances in coffee such as magnesium, trigonelline, and quinides improve insulin sensitivity. Despite the potential harmful effects of caffeine and its ability to release epinephrine (a strong inhibitor of insulin), the aforementioned compounds appear to counterbalance this effect.^39^ In a large prospective cohort study conducted by de Koning Gans et al (2010), the consumption of black tea and coffee was associated with a lower incidence of coronary heart disease mortality.^40^ Andersen et al (2006) reported that consumption of 1 to 3 cups of coffee per day was associated with a lower incidence of cardiovascular disease–related mortality (HR 0.76, 95% CI 0.64 to 0.91, *P*=0.005) in females compared to consumption of >4 cups per day**.**^41^ An earlier study by Kleemola et al (2000) noted a decrease in total mortality among females drinking >7 cups of coffee per day (risk ratio 0.62, 95% CI 0.44 to 0.87), even after adjusting for age, smoking status, serum cholesterol level, blood pressure, and history of myocardial infarction.^42^ Lopez-Garcia et al (2008) found a reduction in RR for all-cause mortality among females: RR of 0.93 (95% CI 0.87 to 0.98) for those drinking 5 to 7 cups per week; RR of 0.82 (95% CI 0.77 to 0.87) for those drinking 2 to 3 cups per day; RR of 0.74 (95% CI 0.68 to 0.81) for those drinking 4 to 5 cups per day; and RR of 0.83 (95% CI 0.73 to 0.95) for those drinking ≥6 cups per day (*P* for trend <0.001).^39^ In fact, most studies show an overall reduction in mortality, although some studies have produced conflicting results. The FHS showed that coffee consumption in the elderly population was associated with a 43% reduction in coronary heart disease–related deaths.^31^ Furthermore, a large meta-analysis (40 studies, n=3,852,651) by Kim et al (2019) revealed that coffee consumption was inversely related to all-cause mortality.^43^ The total number of deaths from all categories was 450,256; among coffee consumers, the intakes with the lowest RRs were 3.5 cups per day for all-cause mortality (RR 0.85, 95% CI 0.82 to 0.89), 2.5 cups per day for cardiovascular disease mortality (RR 0.83, 95% CI 0.80 to 0.87), and 2 cups per day for cancer mortality (RR 0.96, 95% CI, 0.94 to 0.99). The inverse relationship between mortality and coffee consumption in nonsmokers (RR 0.95, 95% CI 0.93 to 0.97) was slightly stronger than in smokers (RR 0.97, 95% CI 0.96 to 0.99), although the difference was not statistically significant (*P*=0.33). No additional benefit was seen with additional caffeine intake. In conclusion, moderate coffee consumption was deemed beneficial to overall health. However, the associations were stronger in Europeans and Asians compared to Americans living in the United States.^43^ Studies are needed to understand these demographic differences.

## DISCUSSION

Coffee has the physiologic effect of raising blood pressure, although the effect appears to be transient and does not affect the propensity to develop overt hypertension. Likewise, data have shown that moderate consumption of coffee is associated with less risk of hypertension but principally in those who never smoked or do not smoke and in those who are fast metabolizers of caffeine. Boiled coffee is atherogenic because of its rich diterpene content, namely cafestol and kahweol, that inhibits bile acid synthesis and ultimately affects lipid metabolism. On the other hand, filtered coffee, which is essentially devoid of the aforementioned compounds, exerts antiatherogenic properties by increasing high density lipoprotein–mediated cholesterol efflux from macrophages through the influence of plasma phenolic acid. As such, cholesterol levels are influenced by the manner of coffee preparation (boiled vs filtered). In terms of the risk of developing cardiovascular disease, studies are inconsistent with regard to coffee consumption and risk of coronary heart disease, with discrepancies noted depending on sex, genetics, and smoking status. The majority of studies showed a J-shape association in which moderate coffee consumption was associated with a lower risk of coronary heart disease compared to heavy coffee consumption which was associated with an increased risk of coronary heart disease. These findings point to the importance of moderate coffee consumption because of the potential risk of cardiovascular disease. Regarding heart failure, results appear to be consistent across studies. The FHS, CHS, and ARIC studies showed that high coffee intake was associated with a decrease in long-term risk of heart failure independent of sex, baseline history of myocardial infarction, and diabetes. Because of the lack of understanding of potential mechanisms behind reduction in heart failure risk, more studies are needed. Moderate coffee consumption was also found to attenuate the risk of atrial fibrillation, possibly owing to its strong anti-inflammatory components such as cafestol, polyphenol, trigonelline, chlorogenic acid, and quinide. Studies have shown moderate coffee consumption to be associated with a reduction in all-cause and cardiovascular disease–related mortality, whereas higher amounts of coffee consumption were detrimental to health.

## CONCLUSION

Our review suggests that moderate coffee consumption is associated with a decrease in all-cause and cardiovascular disease–related mortality, hypertension, cholesterol, heart failure, and atrial fibrillation, while consensus is lacking regarding the association between coffee consumption and the risk of developing cardiovascular disease. Despite previous concerns about coffee consumption being a significant coronary heart disease risk factor, most modern prospective cohort meta-analyses found no association between coffee consumption and coronary heart disease. However, results are inconsistent across studies. Most favorable outcomes are associated with moderate coffee consumption; however, there is no current consensus on the definition of moderate coffee consumption. Further, genetics possibly play a large role in determining hypertension and cardiovascular disease outcomes. This review illustrates that coffee is a complex mixture of compounds that may cause both harm and benefit. As such, additional studies are needed to further elucidate the ideal way to consume coffee, not only as a drink to be enjoyed, but also as a drink with protective health benefits.

## Notes

*This article meets the Accreditation Council for Graduate Medical Education and the American Board of Medical Specialties Maintenance of Certification competencies for Patient Care and Medical Knowledge.*
